# Supplementary material for: Identification of Let-7 miRNA Activity as a Prognostic Biomarker of SHH Medulloblastoma
Source: Cancers (Basel). 2021 Dec 28;14(1):139. doi: 10.3390/cancers14010139 (PMC8750188; doi:10.3390/cancers14010139)
Supplement: Supplementary file 1 [file cancers-14-00139-s001.zip › supplementary figures.pdf]

cancers

Article

Identification of Let-7 miRNA Activity as a Prognostic Biomarker of SHH Medulloblastoma

Maximillian S. Westphal [1], Eunjee Lee [1,2], Eric E. Schadt [1,2], Giselle S Sholler [3,4] and Jun Zhu [1,2,5,*]

1 Sema4, 333 Ludlow St., Stamford, CT 06902, USA; maximillian.westphal@sema4.com (M.S.W.); eunjee.lee@mssm.edu (E.L.); eric.schadt@sema4.com (E.E.S.)
2 Department of Genetics and Genomic Sciences, Icahn School of Medicine at Mount Sinai, 1 Gustave L. Levy Pl, New York, NY 10029, USA
3 Helen DeVos Children's Hospital, Grand Rapids, MI 49503, USA; Giselle.SaulnierSholler@helendevoschildrens.org
4 College of Human Medicine, Michigan State University, Grand Rapids, MI 49503, USA
5 The Tisch Cancer Institute, Icahn School of Medicine at Mount Sinai, 1 Gustave L. Levy Pl, New York, NY 10029, USA
* Correspondence: jun.zhu@mssm.edu

Citation: Westphal, M.S.; Lee, E.; Schadt, E.E.; Sholler, G.S.; Zhu, J. Identification of Let-7 miRNA Activity as a Prognostic Biomarker of SHH Medulloblastoma. *Cancers* **2022**, *14*, 139. https://doi.org/10.3390/cancers14010139

Academic Editors: Gabriella Misso, Angela Lombardi and Agostino Festa

Received: 3 November 2021
Accepted: 23 December 2021
Published: 28 December 2021

Publisher's Note: MDPI stays neutral with regard to jurisdictional claims in published maps and institutional affiliations.

Copyright: © 2021 by the authors. Licensee MDPI, Basel, Switzerland. This article is an open access article distributed under the terms and conditions of the Creative Commons Attribution (CC BY) license (http://creativecommons.org/licenses/by/4.0/).

Supplementary Figures

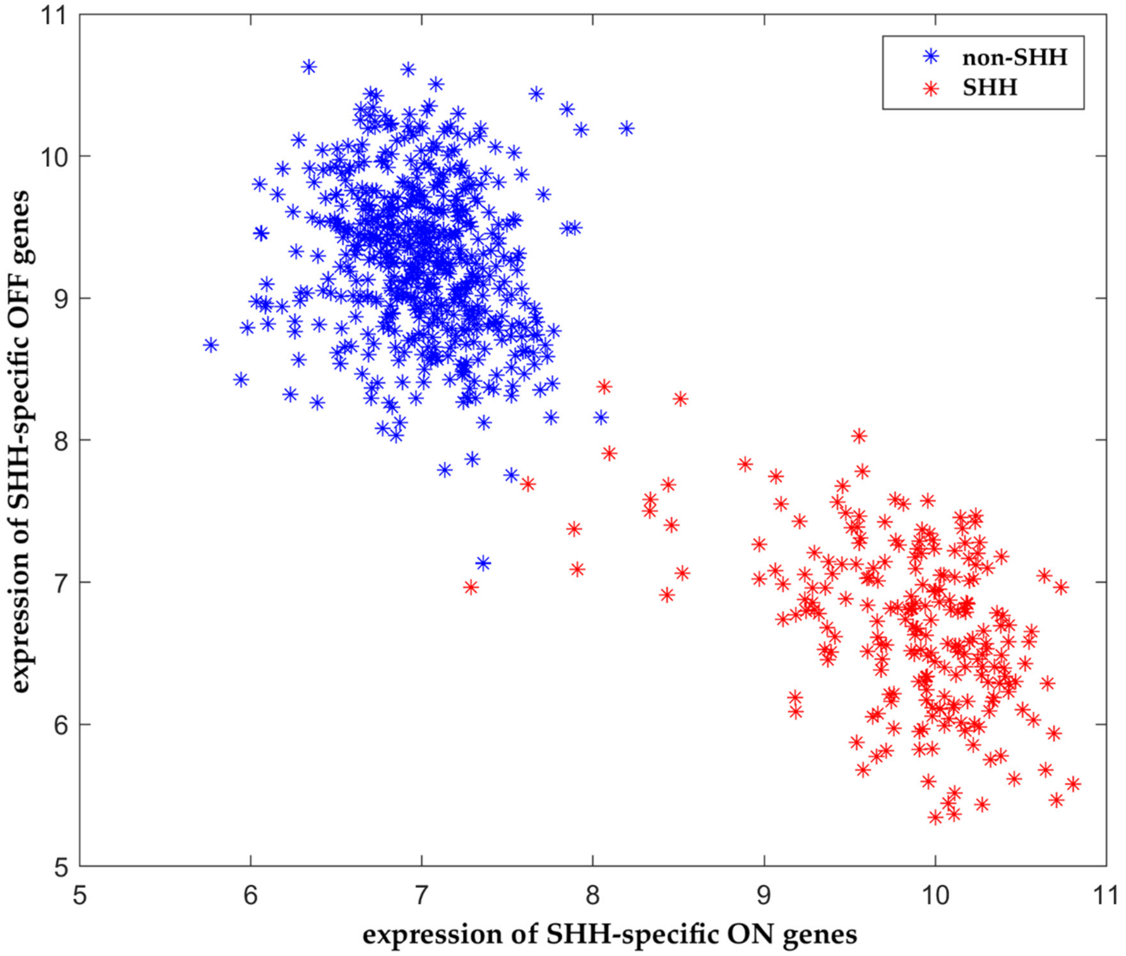

Figure S1. SHH-MB specific genes (On and Off genes) determined using single-cell RNA-seq expression profiles from 25 MB patients in (GSE119926) [55]. Among 589 cis-Methyl genes, 247 and 248 were SHH-MB-specific up/down-regulated with genes expressed higher/lower in SHH-MB than other subgroups at $p < 0.001$, respectively. Within the 247 SHH-MB-specific up-regulated genes, 13 genes were expressed specifically in SHH-MB tumor cell ($p < 0.001$ and expression in non SHH-MB tumor cells < 1.5), referred to as SHH-MB tumor cell specific expressed genes (on genes). Similarly, among 248 SHH-MB specific down-regulated genes, 6 genes did not express in SHH-MB tumor cells specifically ($p < 0.001$ and expression in SHH-MB tumor cells < 1.5), referred as SHH-MB tumor cell specific non-expressed genes (off genes).

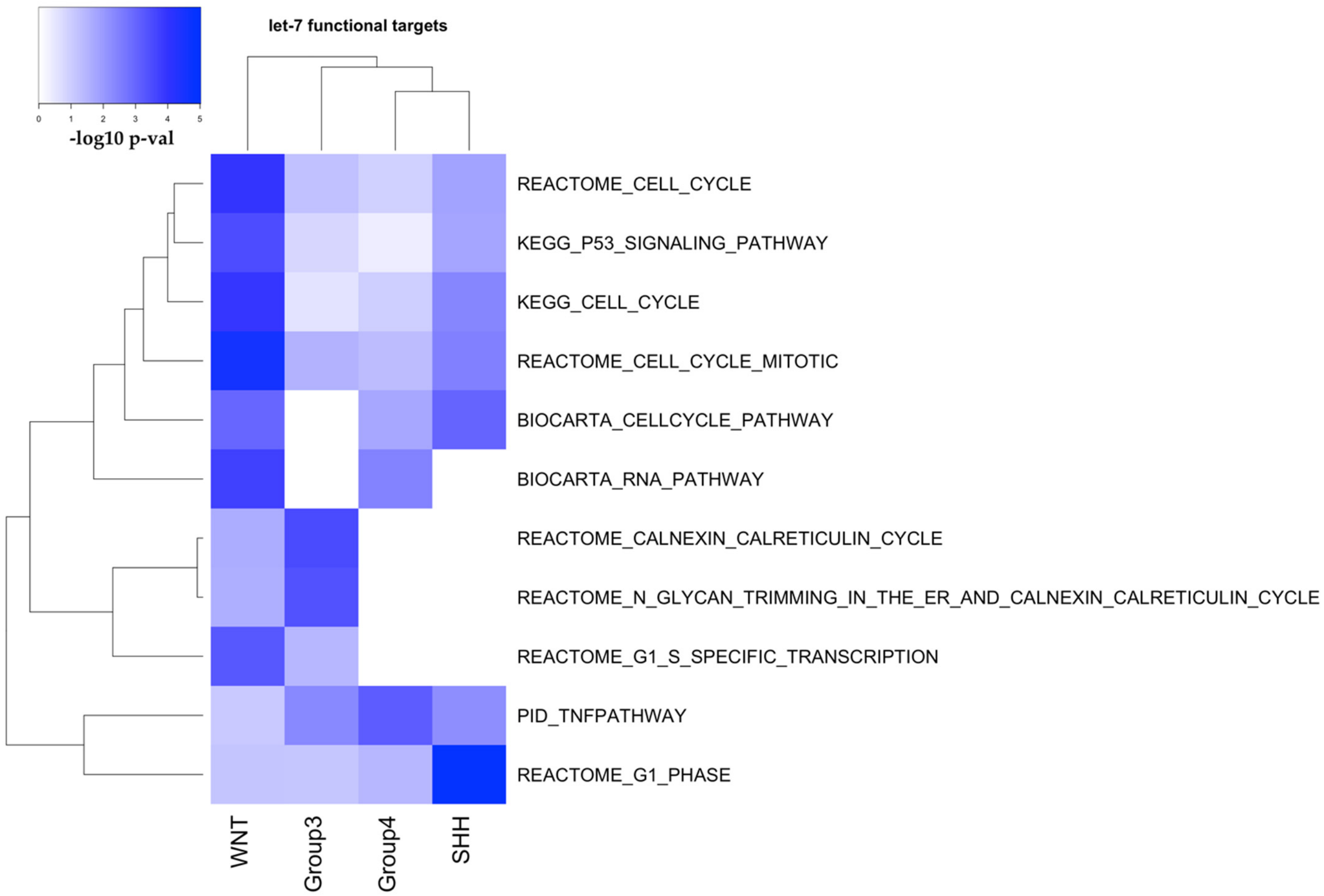

Figure S2. Functional annotation of let-7's functional targets. Heatmap of pathway enrichment of functional target genes of let-7 miRNA for each group of MB samples. The displayed pathways were significantly enriched for target genes of at least one group of MB.

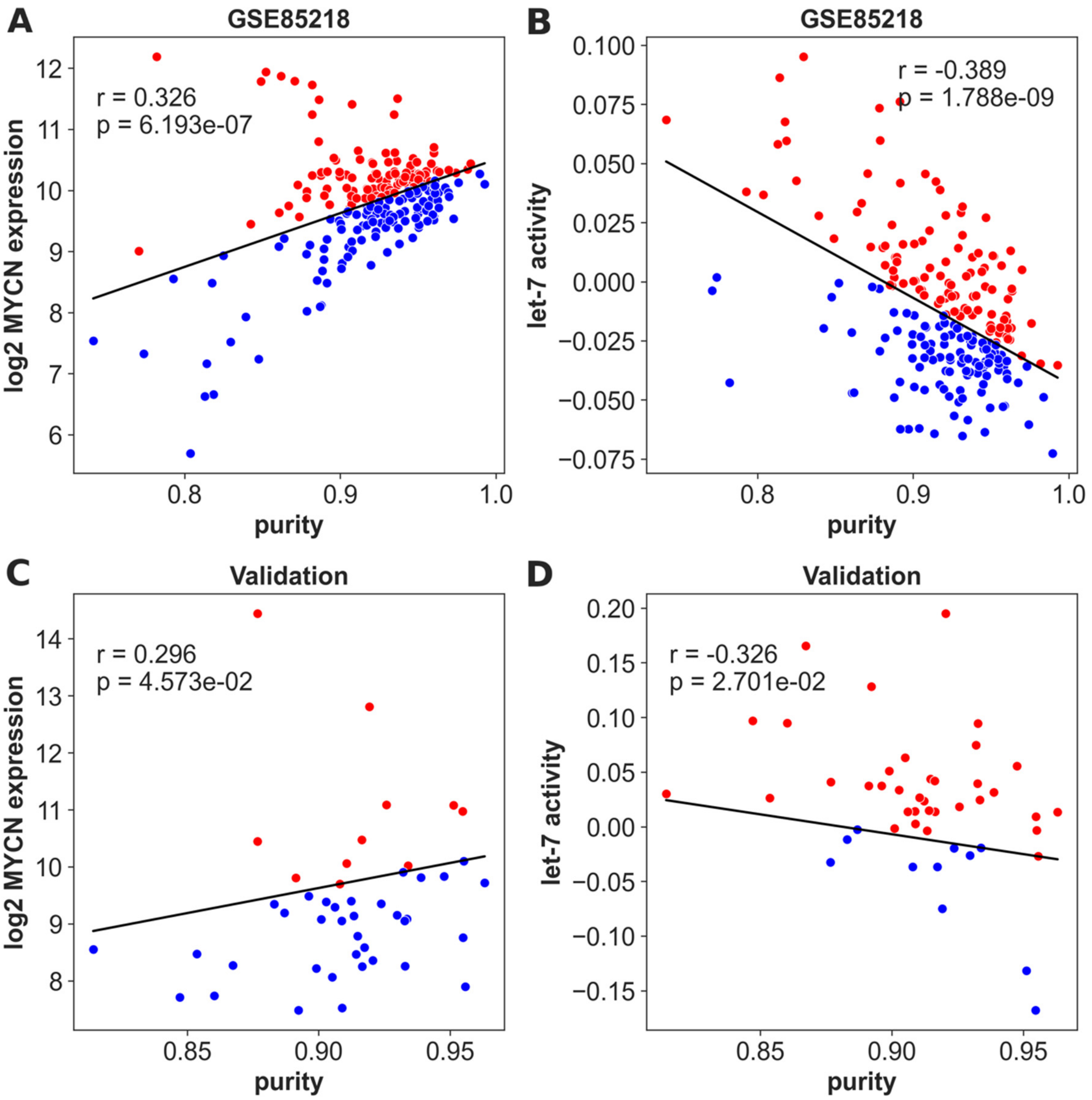

Figure S3. Partitioning SHH-MB samples into MYCN expression low/high or let-7 activity low/high with tumor purity taken into consideration. We performed linear regression between purity (**Methods 2.4)** and MYCN expression and between purity and let-7 activity. The line of best fit (representing mean expression level or activity at a tumor purity level) was used to define high vs low purity. High purity was greater than the linear regression line of best fit, and low purity was defined as less than the linear regression line of best fit. (**A**) GSE85218 linear regression for MYCN vs purity. (**B**) GSE85218 linear regression for let-7 activity vs purity. (**C**): St Jude/validation linear regression for MYCN vs purity**.** (**D**) St Jude/validation linear regression for let-7 activity vs purity.

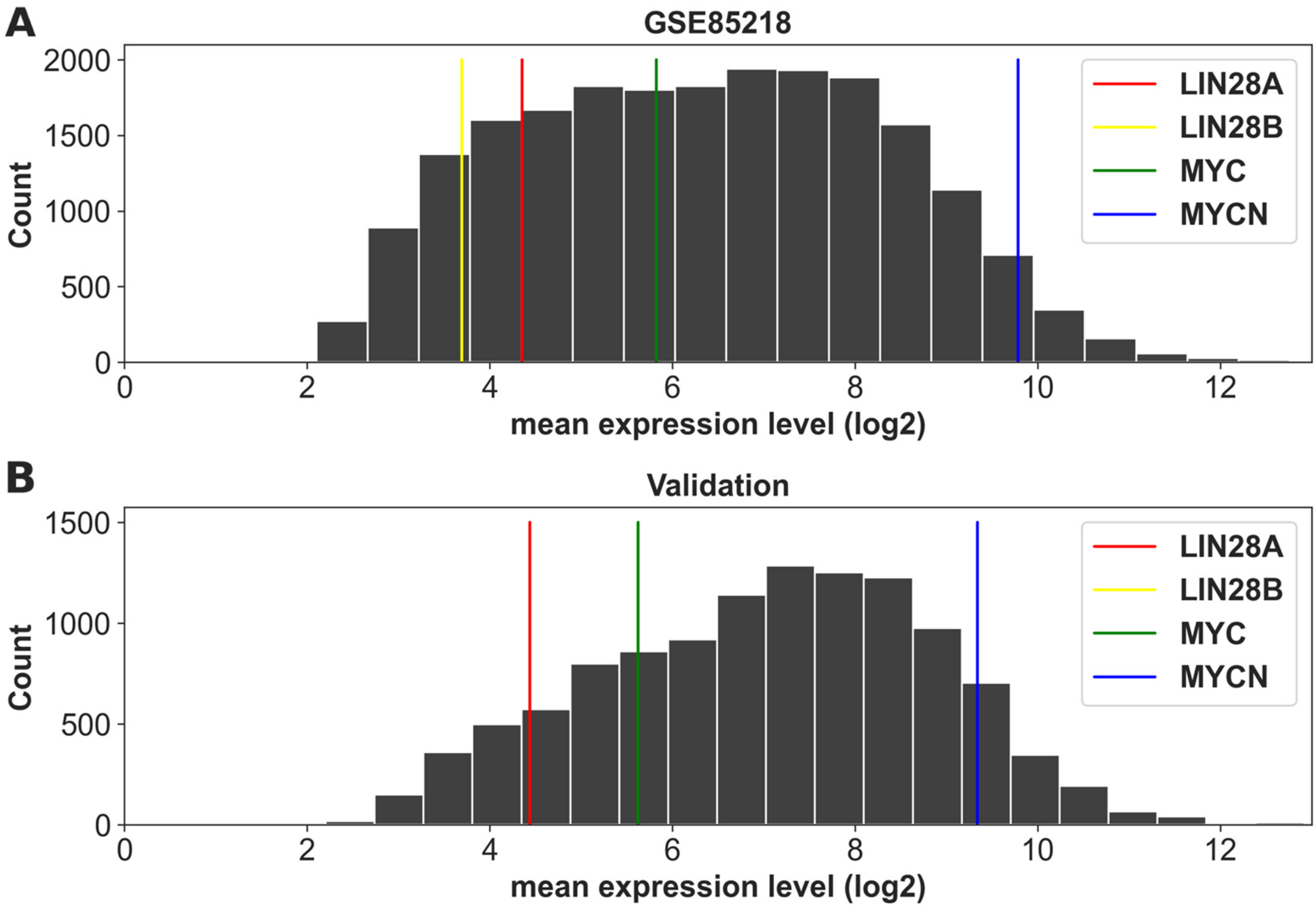

Figure S4. Histogram of mean gene expression levels based on SHH samples in (A) GSE85218 and (B) validation set. Mean expression levels of MYC, MYCN, LIN28A, and LIN28B were indicated as green, blue, red and yellow, respectively.

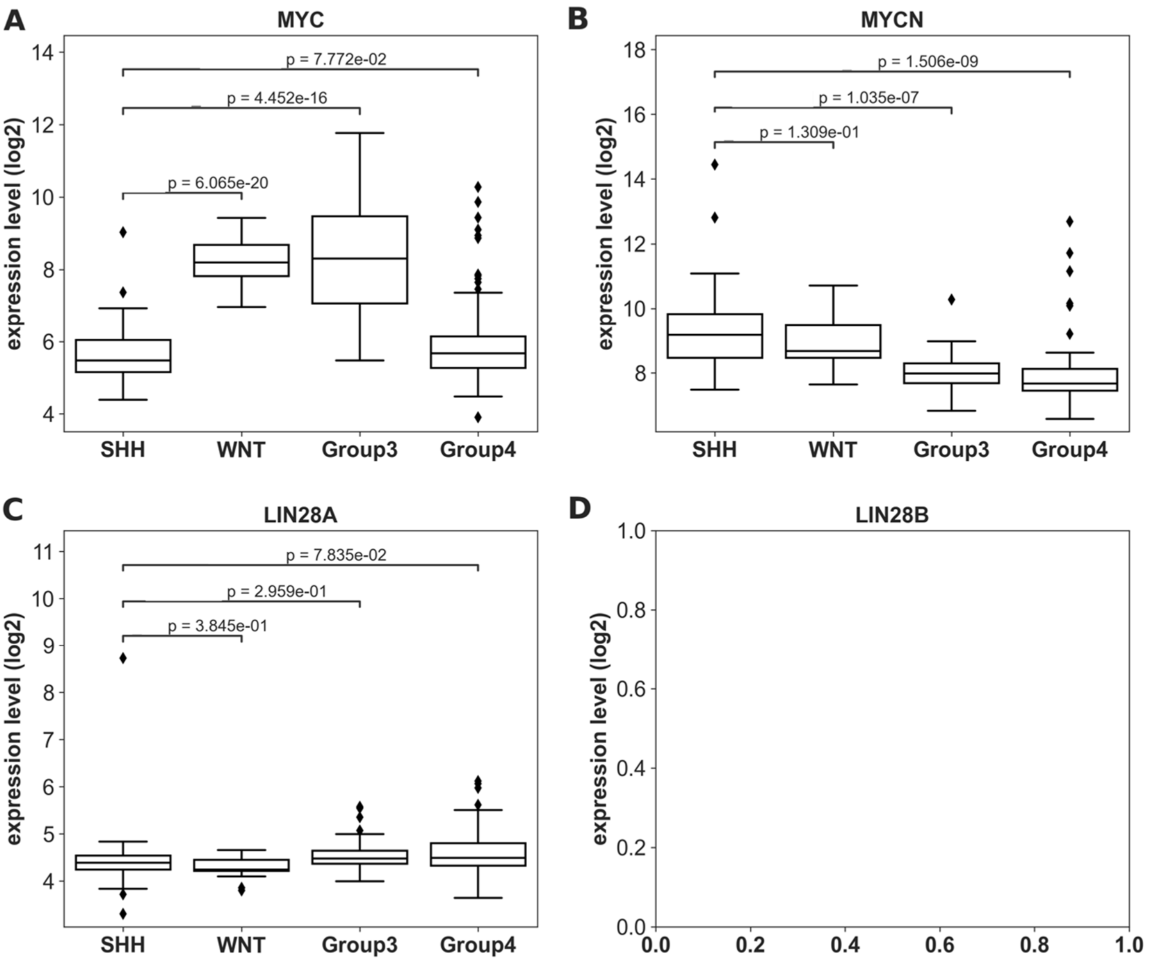

Figure S5. Validation version of Figure 2 based on St. Jude data set. Expression profiles for critical genes in the LIN28 pathway. (**A**–**D**) display the expression distribution of MYC, MYCN, LIN28A, and LIN28B respectively in St. Jude data set. The St Jude data set does not have a column for LIN28B.

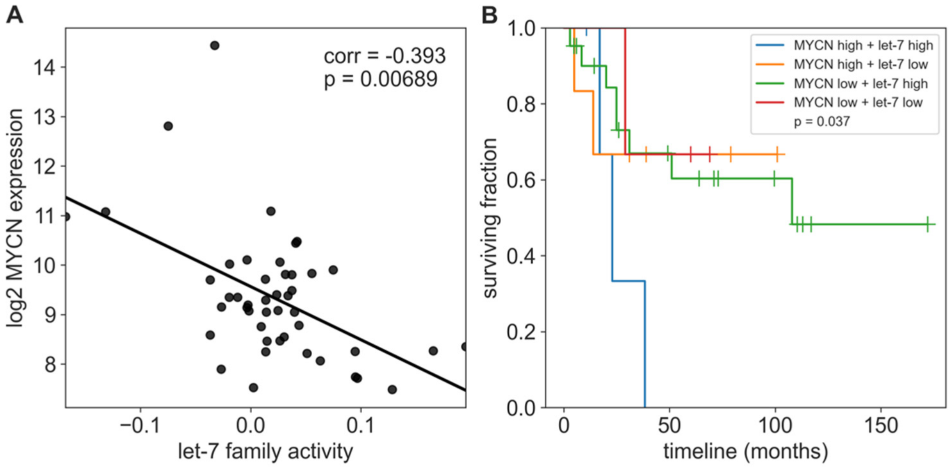

Figure S6. Correlation between MYCN expression and let-7 activity and Kaplan-Meier survival curves for the validation cohort (**A**) the correlation between let-7 family activity and MYCN expression (Spearman correlation γ= -0.393 and p = 0.00689); (**B**) **Figure 7C** “Others” group shown as independent groups.

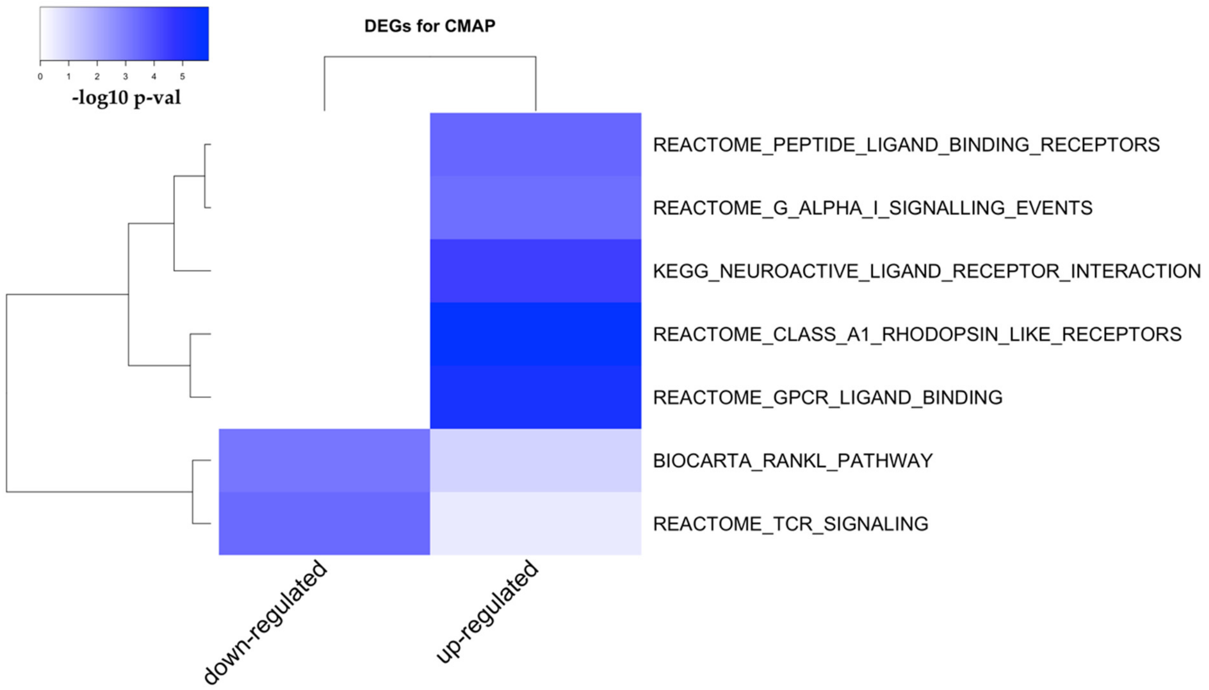

Figure. S7: **Functional annotation of differentially expressed genes (DEGs) used for CMAP.** DEGs were determined by comparing SHH-MB with both high MYCN expression and high let-7 activity against the other SHH-MB samples.

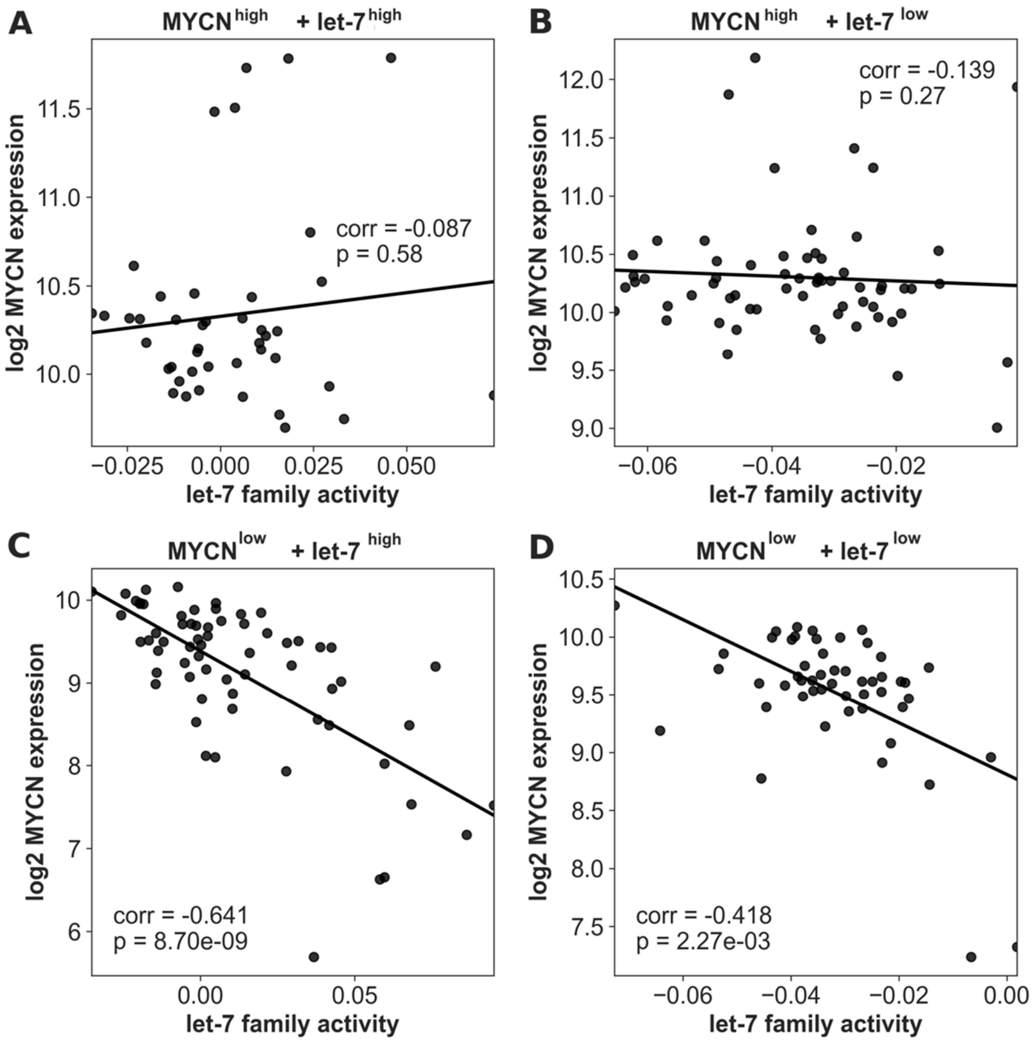

Figure S8. Comparison between let-7 family activity and MYCN expression levels for the four SHH groups separated by MYCN expression and let-7 activity in the validation cohort: (**A**) MYCN high/let-7 high. (**B**) MYCN high/let-7 low. (**C**) MYCN low/let-7 high. (**D**) MYCN low/let-7 low.

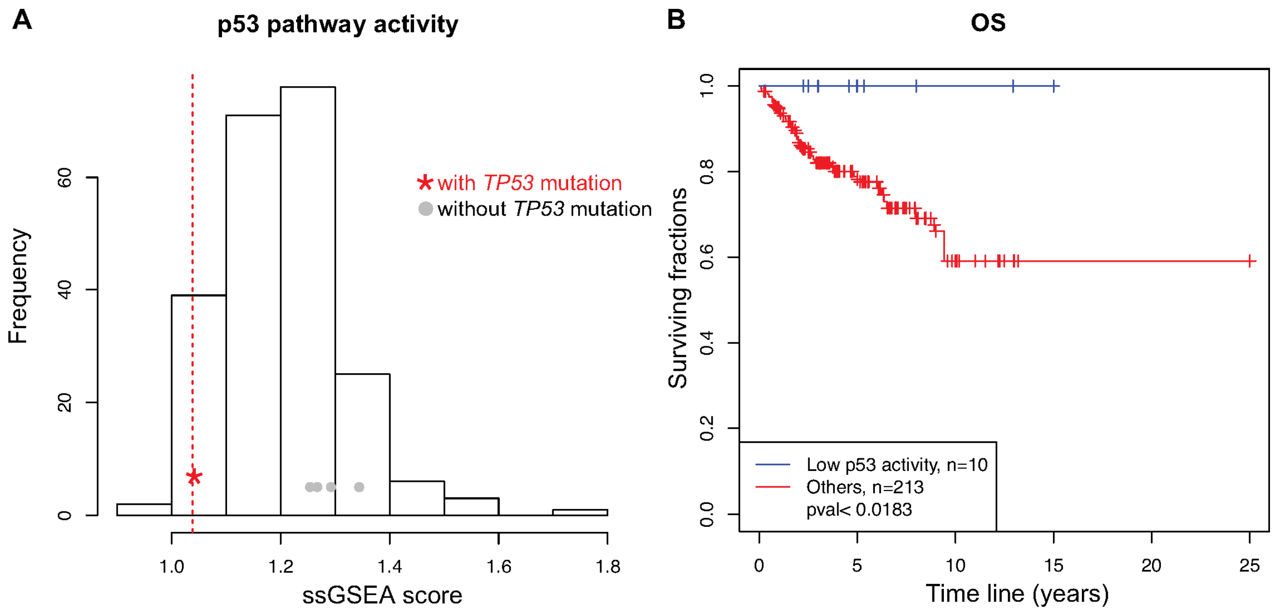

Figure S9. TP53 in SHH-MB samples of GSE85218. (**A**) the distribution of p53 pathway activity in SHH-MB. We applied single sample gene set enrichment analysis (ssGSEA) method in GSVA package [90] by using genes within Hallmark p53 pathways [91]. The red star indicates the sample with known *TP53* mutation status while gray circles indicate without *TP53* mutation. The *TP53* mutation information is based on Northcott et al. [12] data set. The table indicating matched samples between GSE85218 and Northcott et al data was downloaded from Skowron et al. [89]. (**B**) Kaplan-Meier survival curves for samples with p53 pathway activity scores lower than or equal to that of the sample with *TP53* mutation (shown in blue) and the rest of samples (shown in red).

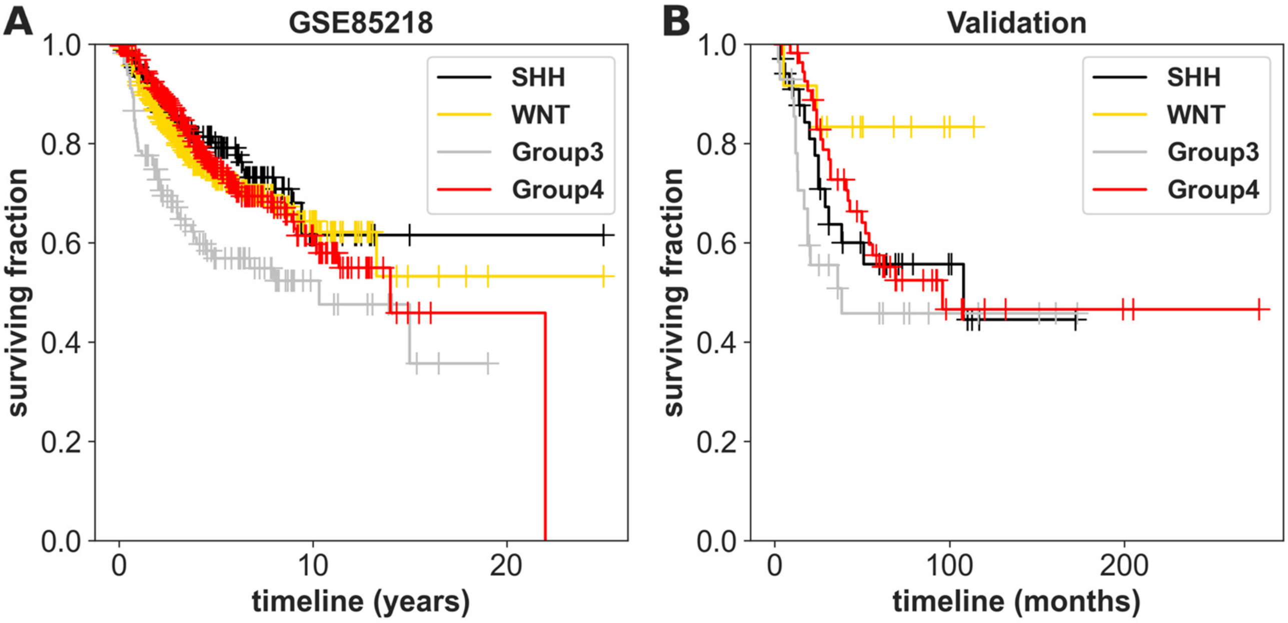

Figure S10. Subgroup KM curve based on (**A**) **GSE85218 and** (**B**) **validation data set.** SHH, WNT, Group3 and Group4 subtypes were indicated as black, yellow, gray and red respectively.

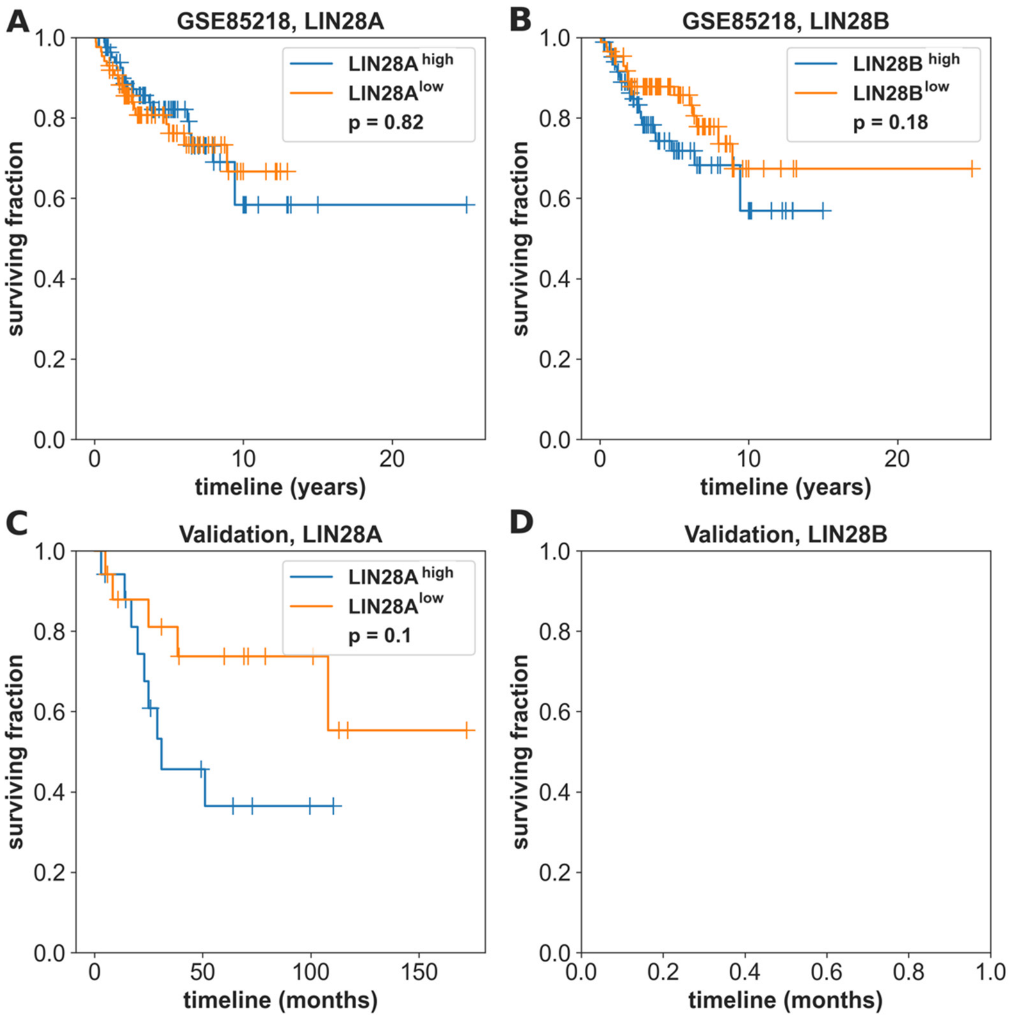

Figure S11. Kaplan-Meier overall survival curves for LIN28A and LINB expression levels based on SHH MB samples of (**A**,**B**) **GSE85218 and** (**C**,**D**) **validation data sets.** High and low LIN28A and LIN28B expression level were determined by median expression levels. The validation dataset did not have a column for LIN28B.

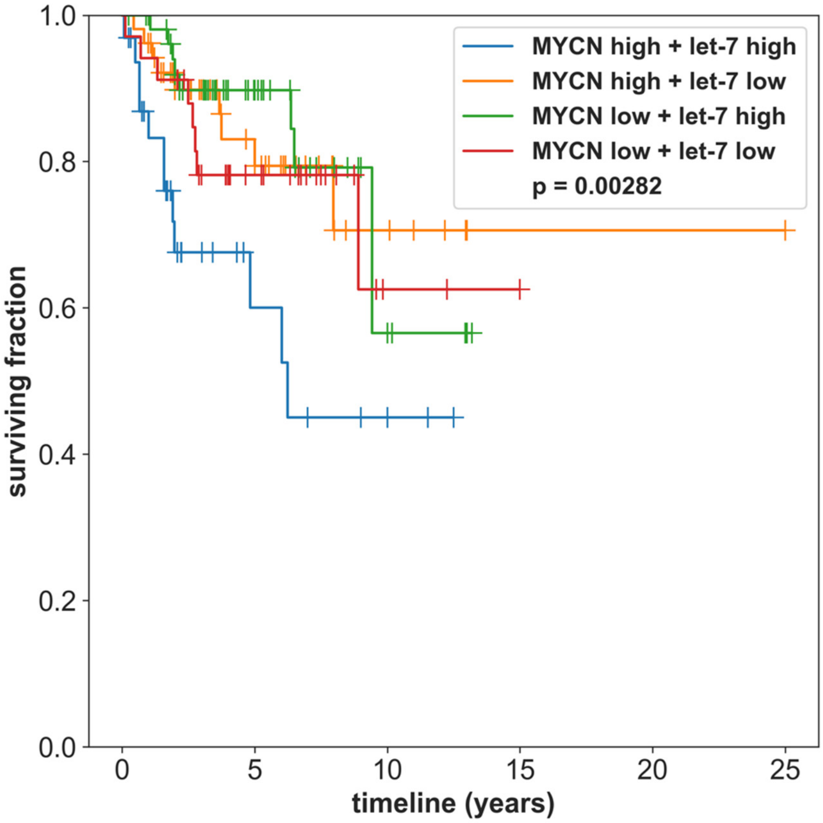

Figure S12. Kaplan-Meier survival curves for GSE85218 displaying individual groups included in "Others" group in the **Figure 6C** .

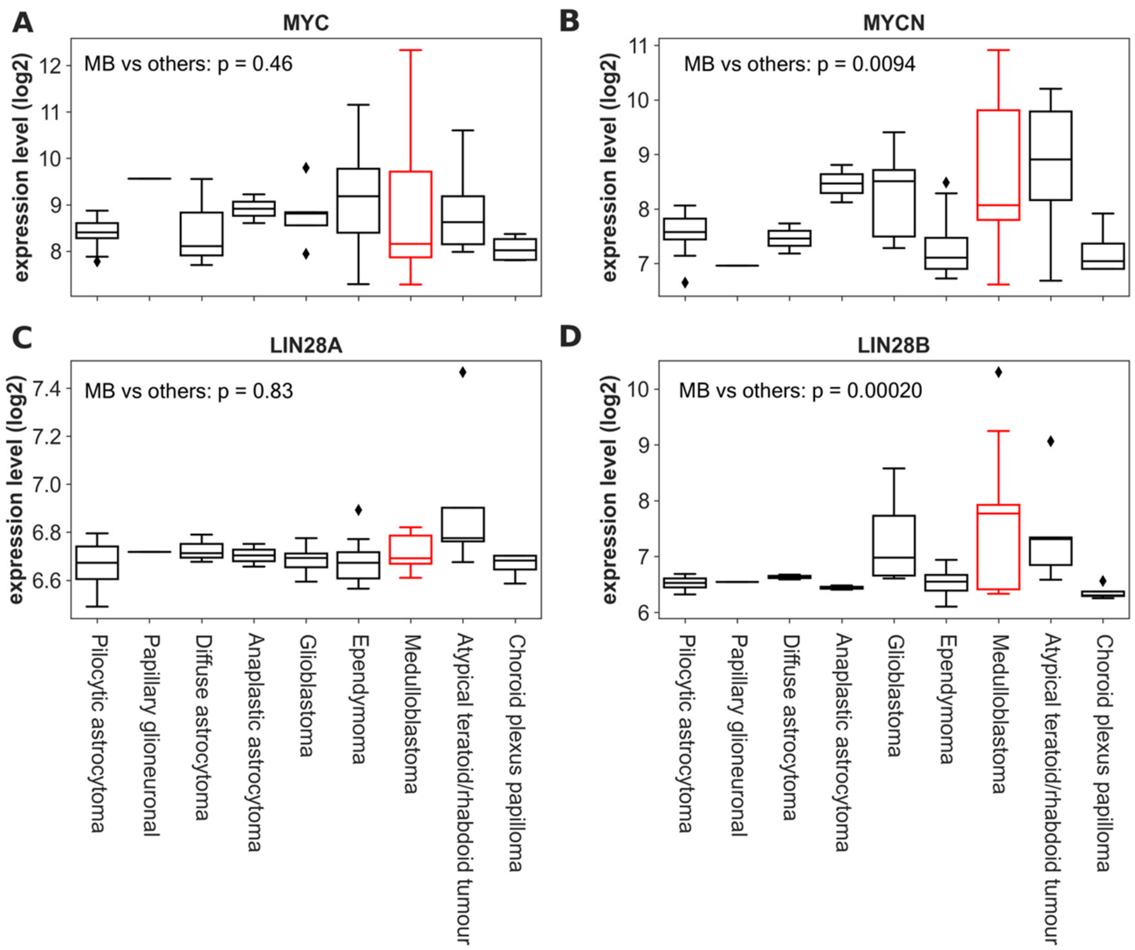

Figure S13. Expression of MYC (**A**), MYCN (**B**), LIN28A (**C**), and LIN28B (**D**) in GSE42658 by tissue. *p*-values displayed are one vs. others *t*-tests between MB and other tissues.
